# Comparison of the Distinct, Host-Specific Response of Three *Solanaceae* Hosts Induced by *Phytophthora infestans*

**DOI:** 10.3390/ijms222011000

**Published:** 2021-10-12

**Authors:** Jie Lu, Tingli Liu, Xiong Zhang, Jie Li, Xun Wang, Xiangxiu Liang, Guangyuan Xu, Maofeng Jing, Zhugang Li, Ingo Hein, Daolong Dou, Yanju Zhang, Xiaodan Wang

**Affiliations:** 1 College of Agriculture, Northeast Agricultural University, Harbin 150030, China; lujie@haas.cn; 2 Department of Plant Pathology, College of Plant Protection, China Agricultural University, Beijing 100193, China; jieli2020@cau.edu.cn (J.L.); liangxiangxiu@cau.edu.cn (X.L.); xuguangyuan@cau.edu.cn (G.X.); ddou@njau.edu.cn (D.D.); 3 Heilongjiang Academy of Agricultural Sciences, Harbin 150028, China; wangxun@haas.cn (X.W.); lizhugang@haas.cn (Z.L.); 4 Excellence and Innovation Center, Jiangsu Academy of Agricultural Sciences, Nanjing 210014, China; liutingli@jaas.ac.cn; 5 Key Laboratory of Biology and Genetic Improvement of Oil Crops, Ministry of Agriculture of the PRC, Oil Crops Research Institute, Chinese Academy of Agricultural Sciences, Wuhan 430062, China; zhangxiong@caas.cn; 6 The Key Laboratory of Plant Immunity, College of Plant Protection, Nanjing Agricultural University, Nanjing 210095, China; jingmf@njau.edu.cn; 7 The James Hutton Institute, Invergowrie, Dundee DD2 5DA, UK; ingo.hein@hutton.ac.uk

**Keywords:** *Solanaceae* hosts, *Phytophthora* *infestans*, RNA-seq, host-specific immune response

## Abstract

Three *Solanaceae* hosts (TSHs), *S. tuberosum*, *N. benthamiana* and *S. lycopersicum*, represent the three major phylogenetic clades of *Solanaceae* plants infected by *Phytophthora infestans*, which causes late blight, one of the most devastating diseases seriously affecting crop production. However, details regarding how different *Solanaceae* hosts respond to *P. infestans* are lacking. Here, we conducted RNA-seq to analyze the transcriptomic data from the TSHs at 12 and 24 h post *P. infestans* inoculation to capture early expression effects. Macroscopic and microscopic observations showed faster infection processes in *S. tuberosum* than in *N. benthamiana* and *S. lycopersicum* under the same conditions. Analysis of the number of genes and their level of expression indicated that distinct response models were adopted by the TSHs in response to *P. infestans*. The host-specific infection process led to overlapping but distinct in GO terms and KEGG pathways enriched for differentially expressed genes; many were tightly linked to the immune response in the TSHs. *S. tuberosum* showed the fastest response and strongest accumulation of reactive oxygen species compared with *N. benthamiana* and *S. lycopersicum*, which also had similarities and differences in hormone regulation. Collectively, our study provides an important reference for a better understanding of late blight response mechanisms of different *Solanaceae* host interactions.

## 1. Introduction

*Solanum*, with approximately 1500 species, is the largest plant family in the *Solanaceae* and includes economically important species, for example, potato (*Solanum tuberosum* L.) and tomato (*Solanum lycopersicum*), as well as model tobacco plants *Nicotiana benthamiana* [1,2]. As the world’s third most important food crop, potato has high nutritional value and plays an important role in enhancing global food security [3]. Tomato is a vital horticultural and economic crop cultivated worldwide [4]. Meanwhile, *N. benthamiana*, has been widely employed for studies of host-pathogen interaction due to its susceptibility to many relevant pathogens [5,6]. Despite being relatively closely related species, the genomes of the three *Solanaceae* hosts (TSHs) described above display sequence differentiation in phylogenetic tree, *S. tuberosum* and *S. lycopersicum* are closed wherever *N. benthamiana* relatively far away [7,8,9]. These three plants also have host specific traits, for example, tomato is the sole host of *Cladosporium fulvum* [10]. These sequence differentiations and host specific traits may trigger specific phenotypic and immune responses when attacked by various pathogens.

Late blight caused by the hemibiotroph oomycete *Phytophthora infestans* is one of the most devastating crop diseases [11,12]. However, the host range of *P. infestans* is relatively narrow and mainly limited to *Solanaceous* crops, such as potato and tomato. Epidemically, *P. infestans* causes stem, leaf, fruit and tuber rot, largely affects crops’ yield and quality leading to significant economic losses [13,14]. Under cool, wet conditions, *P. infestans* can infect and produce thousands of sporangia from a single lesion that easily become air-borne, resulting in prolific spread of the pathogen and entire crops can be destroyed within days. Due to the serious threat in production value of economical crops, late blight of *Solanaceae* species has always been the focus of in-depth research [15,16,17].

In most cases, the plant immune system can effectively sense and respond to pathogens’ colonization and restrict majority growth of the pathogens [18]. Understanding how plant diseases occur and how the plant’s immune system effectively responds or restricts pathogen infection is critical to controlling those diseases. The early stages of infection are arguably the most critical for hosts to quickly respond to pathogen infection [19]. Many reports have studied plant innate immunity, downstream immune signaling elements and their modes of action in plant cells [20]. In the early stage, one of the most important immune responses is a rapid burst of reactive oxygen species (ROS), which is critical for successful activation of immune responses of plants against pathogen infection [21,22,23]. Moreover, ROS plays function to induce innate immune responses, including pattern-triggered immunity (PTI) and effector-triggered immunity (ETI) [24]. Next, downstream signaling encompasses mitogen-activated protein kinase (MAPKs) cascades, calcium influx, calcium dependent protein kinases (CDPKs) pathways, transcriptional reprogramming and phytohormone regulation, etc. [25,26,27]. Then the signal transduction network will control salicylic acid (SA)-, jasmonate (JA)- and ethylene (ET)-mediated plant responses to pathogen attack [28,29,30,31,32].

Previous transcriptome studies have documented how foliage defenses against the late blight pathogen are initiated in the TSHs separately [18,33]. Some reports have documented how foliage defends against *P. infestans* separately. Restrepo et al. used the microarray method to examine the interaction of potato leaf against *P. infestans*, highlighting a possible role for carbonic anhydrase (CA) in defining the interaction [34,35]. Moreover, in the aspect of tissue specificity, the differences and similarities of defense mechanisms against *P. infestans* were compared between *S. tuberosum* foliage and tubers [36]. Meanwhile, a large number of sequencing analyses have focused on host-pathogen interactions. For example, the transcriptional dynamics of *P. infestans* in a compatible interaction with tomato showed a tight temporal regulation of many pathways associated with the suppression of plant defense mechanisms and pathogenicity at different infection stages [34]. However, little is known about the differences between the responses of the TSHs to *P. infestans* infection and how the TSH transcriptomes change and are regulated via host-specific responses.

Here, we systematically analyzed the responses of three *Solanaceae* hosts represented by the TSHs to *P. infestans* infection by combining the transcriptome profile with infection phenotypes and investigating the similarities and differences at the early infection stage, at 12 and 24 h post infection (hpi). Following the determination of the gene expression spectrum of *Solanaceae* hosts, the immune regulation of different *Solanaceae* hosts was comprehensively analyzed in terms of ROS accumulation, calcium dependent protein kinase (CDPK) signaling pathways, hormone changes and critical genes. These results revealed distinct expression patterns of the immune-responsive genes in *Solanaceae* hosts to *P. infestans* in the early stage. This study provides novel data for understanding and, in the future, improving the ability of *Solanaceae* crops to resist late blight by realizing targeted and accurate disease resistance strategies at the transcriptional level.

## 2. Results

### 2.1. Comparisons of Infection Process among TSHs

**Figure 1:**
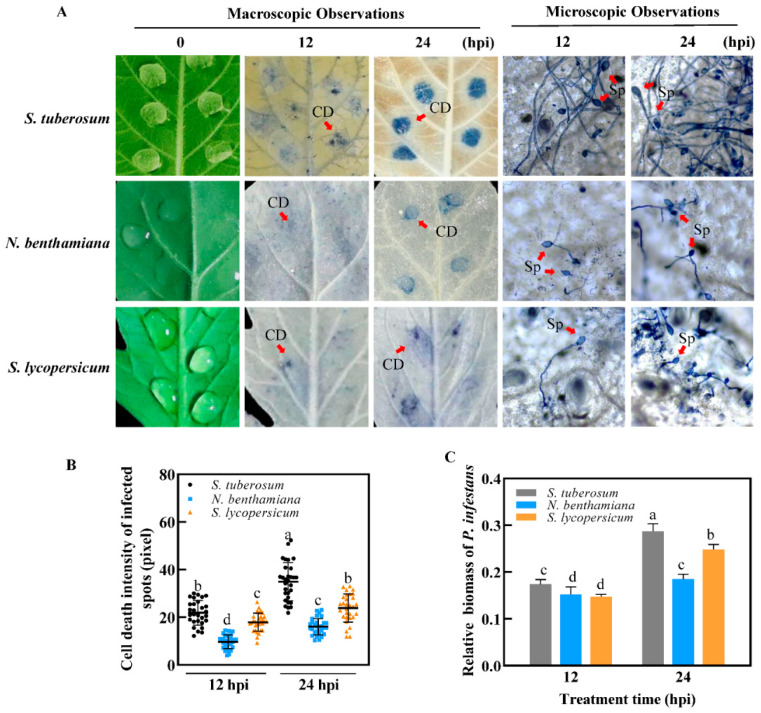
Phenotype of three *solanaceae* hosts (*S. tuberosum*, *N. benthamiana* and *S. lycopersicum*, TSHs) infected by *Phytophthora infestans*. (**A**) Comparisons of *P. infestans* infection on the leaves of TSHs were confirmed by macroscopic and microscopic observation at 0, 12, 24 h post inoculation (hpi). At 12 dpi, cell death (CD) stained by typan blue to small slight blue spots were observed on *S. tuberosum* leaf, but only very small and few blue spots could be macroscopically observed on the leaf of *N. benthamiana* and *S. lycopersicum* compared to *S. tuberosum*. The infection site turned to dark blue stained by typan blue, obvious damage could be observed on *S. tuberosum* leaf at 24 dpi. At 12 hpi, *P. infestans* infection was observed in cells of the TSH. Young hyphae broadly spread formed on the leaves of TSHs, *N. benthamiana* showed the fewest hyphae compared to *S. tuberosum* and *S. lycopersicum* at 24 hpi. More numbers of sporangia (Sp) were microscopically observed on TSHs’ leaves by microscopic observation compared to 12 hpi. (**B**) The relative intensity of cell death on infected spots was quantified by ImageJ software. Each host collected 30 infected spots for observation. (**C**) Relative biomass was calculated by the ratio of Actin DNA of *P. infestans/*plant tissue extracted from diseased leaves in TSHs at 12 and 24 hpi individually via semi-quantitative PCR. Actin DNA value was measured from agarose gel bands after transformation to greyscale images quantified by ImageJ software. Leaf disc samples used for extracting DNA were mixed with 20 inoculation spots. Lower-case letters indicated statistically significant differences (*p* < 0.05, ANOVA). The experiments were repeated three times.

Conditions for examining gene expression were established on the leaves of the TSHs inoculated with *P. infestans* as described in Section 4.1. To further study the differences among the TSHs in response to infection, leaves inoculated with zoospores of *P. infestans* at 0, 12, and 24 hpi were continuously harvested and used to prepare samples for subsequent RNA library construction of early infection process detection in planta. Trypan blue staining is always used as a method to observe cell death, which indicates the infection process of pathogens [37,38]. In order to compare the differences of infection process induced by *P. infestans*, macroscopic and microscopic observations were used to monitor the disease development process (Figure 1A and Figure S1).

From a macroscopic view of the phenotype, at 12 hpi, small light blue spots stained by trypan blue were macroscopically observed on the inoculation site of leaves of the TSHs. At 24 hpi, the infection sites turned dark blue, and obvious damage could be observed in the TSHs. Cell death at the infection site on the three *Solanaceae* hosts (TSHs) was stronger at 24 hpi than at 12 hpi, which was confirmed by the gray scale of the cell death intensity from macroscopic observation (Figure 1B). *S. tuberosum* showed the strongest cell death induced by *P. infestans* compared to both *N. benthamiana* and *S. lycopersicum*, especially at 24 hpi. No macroscopic differences were observed between *N. benthamiana* and *S. lycopersicum*. Cell death caused by *P. infestans* in *N. benthamiana* is the smallest compared to the two other hosts.

From a microscopic view of the phenotype, when studied using light microscopy at a low resolution, at 12 hpi, *P. infestans* infection was observed in cells of the TSH (Figure 1A and Figure S2). Young hyphae broadly spread into and formed haustoria, which was a hallmark of pathogen invasion success during early infection at the biotrophic stage of *P. infestans* growth and sporulation [36]. These haustoria formed on the leaves of the TSHs. At 24 hpi, expanded hyphae appeared on the leaves of the TSHs, where *S. tuberosum* had quickly hyphae expand compared to *S. lycopersicum* and *N. benthamiana*. More hyphae were microscopically observed on the leaves of the TSHs at 24 hpi than at 12 hpi. *N. benthamiana* showed the fewest hyphae compared to *S. tuberosum* and *S. lycopersicum* at 24 hpi via examination of relative biomass (Figure 1C and Figure S3). Therefore, the results indicated that the infection process was obviously different among the leaves of the TSHs infected with *P. infestans* both macroscopically and microscopically at 12, and 24 hpi, and *S. tuberosum* was more rapidly infected than *N. benthamiana* and *S. lycopersicum*.

### 2.2. Initial Analysis of the Infection-Based Transcriptome of Diseased TSHs

**Figure 2:**
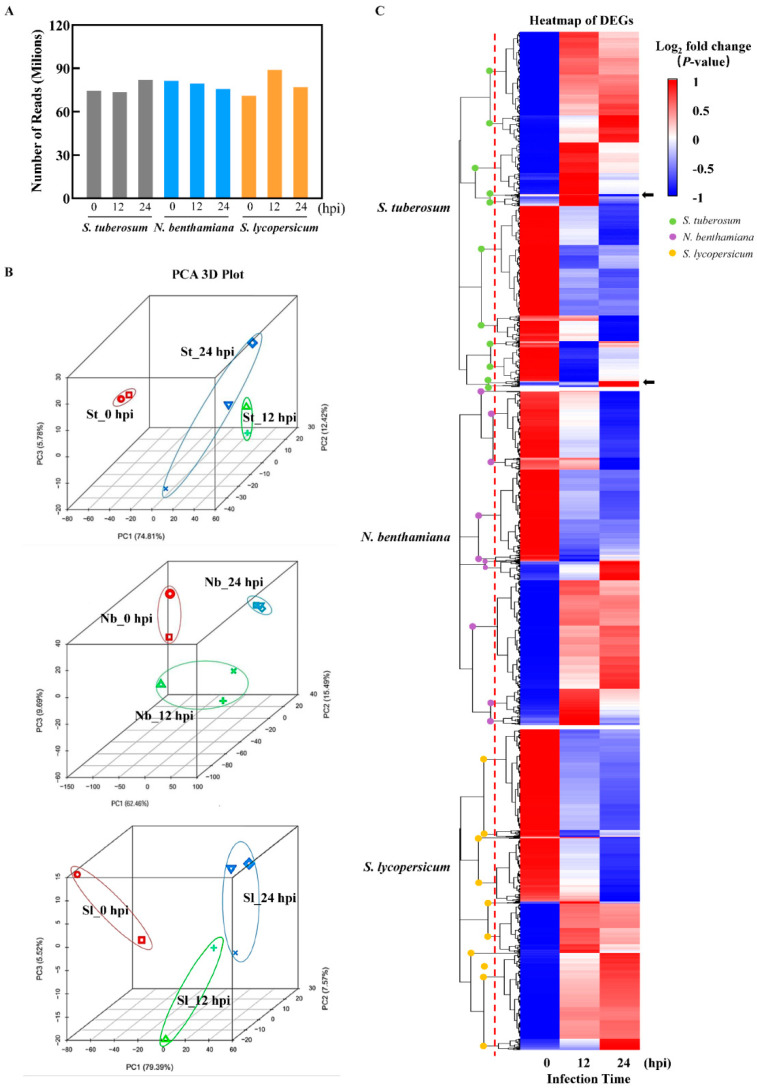
Summary of the initial analysis for the transcriptome data. (**A**) High-quality clean reads from high-throughput sequencing. Total leaf RNA from *S. tuberosum*, *N. benthamiana* and *S. lycopersicum* infected by *P. infestans* at 0, 12 and 24 hpi were used to prepare the high-throughput sequencing library. (**B**) Principal component analysis of differentially expressed genes was employed to visualize the distinction between TSHs infected by *P. infestans* at 0, 12 and 24 hpi. The upper, middle and bottom parts are *S. tuberosum* (St), *N. benthamiana* (Nb) and *S. lycopersicum* (Sl), respectively. The same color represented the repetition of the sample. (**C**) The heat-map of DEGs of TSHs induced by *P. infestans*. The color scale showed the values (log2 fold change). The red dotted-lines represented the locations where TSHs were compared in cluster numbers, and the clusters were compared using different colors dots with green (*S. tuberosum*), purple (*N. Benthamiana*) and orange (*S. Lycopersicum*).

To obtain the spectrum of gene expression of TSHs in response to the infection by *P. infestans*, transcriptome data analysis was conducted for the leaves of TSHs inoculated by *P. infestans* at 0, 12 and 24 h. In biological replicates, RNA-seq was performed for each species and each time point to obtain twenty-two samples in total. After data filtering and quality assessment, approximately 8 Gb of clean Illumina sequencing data were produced, yielding a range of clean bases per sample (Figure 2A). The Q30 percentage (sequencing error rate <0.1%) was above 92% (Table S1). Mapping the RNA-seq reads to each species host genomics network, respectively, resulted in mapping percentage above 80% in all cases, attesting to the high quality of the RNA-seq reads and the reference three genomes. The result of principal component analysis (PCA) of the RNA-Seq datasets showed that the first principal components (PC)1, PC2 and PC3 explained 74.81%, 12.42% and 5.78% of the total variation in potato, respectively. In *N. benthamina*, PC1, PC2 and PC3 explained 62.46%, 15.49% and 0.69% of the total variation, respectively. In tomato, PC1, PC2 and PC3 explained 79.39%, 7.57% and 5.52% of the total variation. The PCA 3D plots show that the infected samples showed that the infected samples of each host were clustered together according to time points, also the clustering at different time points is relatively independent. Meanwhile, the clustering positions of the TSHs at the same time were different. The results indicated that the TSHs had different response modes to pathogen infection. (Figure 2B). Hierarchical clustering was performed for differentially expressed genes on the basis of their *p*-values, and the clustering is shown in Figure 2C. From a general view, half of the DEGs in each host were up-regulated and half were down-regulated. However, for different hosts, there are differences in the clustering of DEGs. For example, when comparing the location of the dotted line, *S. tuberosum*, *N. benthamina*, and *S. lycopersicum* were clustered into 11, 10 and 9 different clusters with different numbers. In addition, some clusters showed specific expression patterns in species. For example, in *S. tuberosum*, the up-regulation ratio of 12 h was higher, and 24 was significantly down-regulated. The three hosts showed different response patterns.

### 2.3. Response Model and Transcript Level of P. infestans Varies with the Solanaceae Host

**Figure 3:**
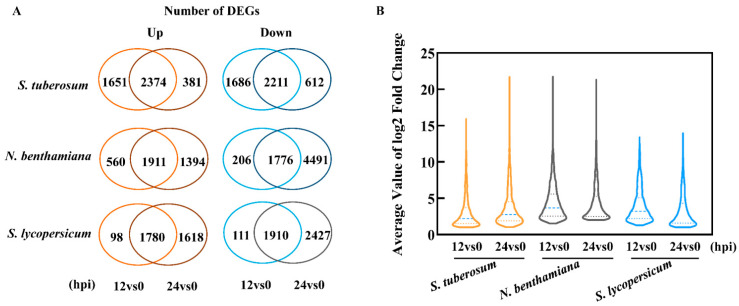
Different expressed genes induced by *P. infestans* among the TSHs. (**A**) Venn diagram representation of the host-specific up-regulated genes distribution at 12 and 24 hpi and the number of up-regulated and down-regulated DEGs induced by *P. infestans* in TSHs (DESeq2 padj ≤ 0.01, |log2 Fold Change| ≥1.0). The upregulated transcripts were shown in orange color, and the downregulated transcripts were shown in blue color. (**B**) The average value of log2 fold change of the host-specific up-regulated genes 12 and 24 hpi. The thick blue dotted line represented the median.

The total number of differentially expressed genes (DEGs) in the diseased TSHs at different time points was presented in Figure 3A. For potato, 7922 and 5578 DEGs were detected, among which 4025 and 2755 genes were upregulated, 3897 and 2823 genes were downregulated at 12 hpi and 24 hpi, respectively. For *N. benthamiana*, 4453 and 9572 DEGs were detected, including 2471 and 3305 upregulated genes, 1982 and 6267 downregulated genes, at 12 hpi and 24 hpi, respectively. For *S. lycopersicum*, 3899 and 7735 DEGs were detected, including 1878 and 3398 upregulated genes, 2021 and 4337 downregulated genes, at 12 hpi and 24 hpi, respectively. The results showed that a higher number of DEGs were expressed during the early stage in *S. tuberosum* at 12 hpi than that in *N. benthamiana* and *S. lycopersicum*, indicating that *S. tuberosum* had the fastest response rate during the early stage of pathogen infection.

A Venn diagram of the transcripts showed that the number of DEGs changed in the TSHs was distinct at different time points (Figure 3A). The most significant differences were the numbers of genes and their expression levels for different hosts in response to *P. infestans*. In *S. tuberosum*, a large number of genes were differentially expressed in response to infection at 12 hpi, then declined at 24 hpi. However, in *N. benthamiana* and tomato, there was an increase in the number of DEGs at 24 hpi compared with those at 12 hpi. The statistical results of the average fold change of the upregulated genes were increased in diseased *S. tuberosum* at 24 dpi over 12 dpi, but a different situation was shown in *N. benthamiana* and *S. lycopersicum* (Figure 3B). In total, the speed of response to *P. infestans* was faster in *S. tuberosum* than in *N. benthamiana* and *S. lycopersicum* at the early infection stage, they adopted different modes in response to *P. infestans*.

### 2.4. Distinct Response Influenced by Different Speed of Infection Process among TSHs

The DEGs screened in the 6 comparison groups St_12-vs-St_0, St_24-vs-St_0, Nb_12-vs-Nb_0, Nb_24-vs-Nb_0, Sl_12-vs-Sl_0 and Sl_24-vs-Sl_0, were subjected to GO and KEGG enrichment analyses. Based on the DEGs functional annotation via GO term enrichment, the results showed that *Solanaceae* hosts mainly respond to *P. infestans* through upregulation rather than downregulation of immunity-related genes (Table S2). Indeed, the biological process (BP) GO terms that are closely related with defense response function are enriched in the induced genes at 12 hpi and 24 hpi (*p* value < 0.05). The induced DEGS can be divided into four main categories: signal transduction, defense response, localization and transport.

**Figure 4:**
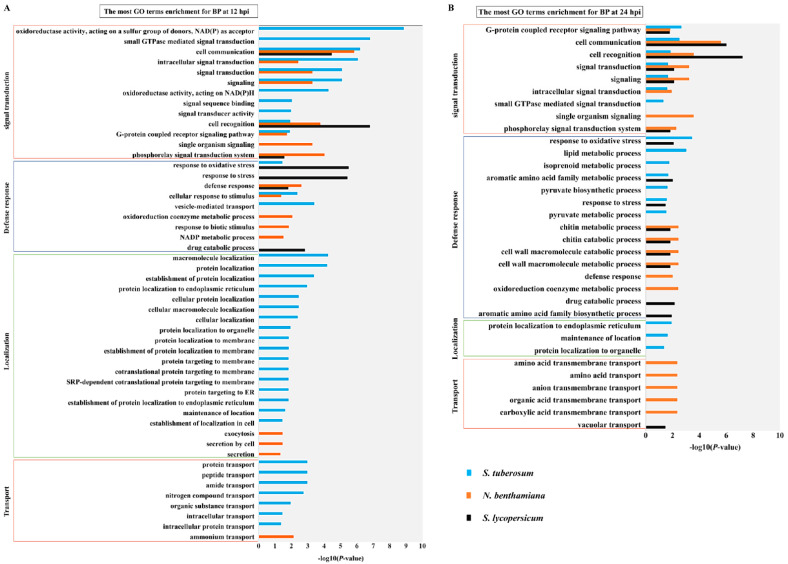
Analysis of biological process in TSHs related response induced by *P. infestans*. (**A**) GO annotation of identified response-related genes in biological processes (BPs) for TSHs at12 hpi; BP terms comparation among TSHs are represented by histogram. The horizontal axis was derived from their log10 (*p*-value) (adjusted *p*-value < 0.05), vertical axis represented GO terms. (**B**) GO annotation of identified response-related genes in biological processes (BPs) for TSHs at 24 hpi. Within each host analysis, the blue bar represented potato, the orange bar represented *N. benthamiana* and the black bar represented tomato.

A comparison of the BP categories among the TSHs at 12 hpi showed that *S. tuberosum* is enriched in the four categories noted above, while *N. benthamiana* and *S. lycopersicum* are enriched only in signal transduction and defense responses (Figure 4A). A comparison of the biological process categories among the TSHs at 24 hpi manifested that *S. tuberosum* and *N. benthamiana* are enriched in all four categories, while *S. lycopersicum* was still only enriched in signal transduction and defense responses (Figure 4B). These results suggested that different hosts relied on different infection processes under the same time and infection conditions, and transcriptional changes in *S. tuberosum* are more pronounced at the early timepoints compared to *N. benthamiana* and *S. lycopersicum*. In contrast to the BP categories among the TSHs at 12 hpi and 24 hpi, the results revealed that there are similarities among the TSHs as, for example, all TSHs activated the signal transduction and defense response. Nevertheless, the differences among the TSHs were that *S. tuberosum* activated localization and transport at multiple sites in cells within 12 h, which was earlier than the other two plants.

In detail, in terms of signal transduction, TSHs all enriched in cell communication (GO:0007154) and cell recognition (GO:0008037), which indicated that cell recognition and signal transmission play an important role in responding to *P. infestans* infection. More interestingly, *S. tuberosum* was specifically enriched in Go terms that closely related to the production of reactive oxygen species, suggesting that *S. tuberosum* activated many more ROS production genes than *N. benthamiana* and *S. lycopersicum* in response to successful *P. infestans* infection (Figure 4A). In terms of defense response, the GO terms enriched among the TSHs were significantly different (Figure 4A). In terms of localization, *S. tuberosum* significantly changed in cell membrane systems. At the same time, a large number of protein transport occurred in *S. tuberosum*, indicating that the *S. tuberosum* cells underwent drastic and complex changes in response to *P. infestans* infection.

In the comparative analysis of BP categories among the TSHs at 24 hpi, *S. tuberosum* and *N. benthamiana* were enriched in all four categories, while *S. lycopersicum* was enriched only in the signal transduction and defense response categories (Figure 4B). In conclusion, due to the different levels of transcriptional response in different hosts under the same infection conditions, *S. tuberosum* activated kinds of response, secondary metabolism processes and protein locations that may indicated *S. tuberosum* had more pronounced of transcriptional changes at the early timepoints compared to *N. benthamiana* and *S. lycopersicum* in response to *P. infestans* infection. However, *S. lycopersicum* may be at an early station level for PTI response to *P. infest ans*. The results suggested that the infection process of *P. infestans* in *Solanaceae* hosts presented is somewhat host-specific.

**Figure 5:**
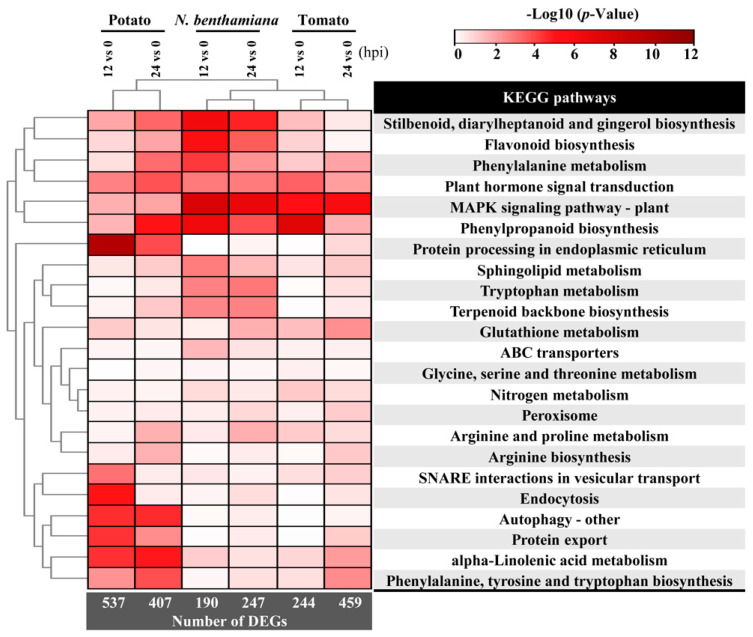
Hierarchical clustering analysis of KEGG pathways comparison demonstrated the differential response among TSHs induced by *P. infestans*. DEGs related to significant enrichment analysis were involved in different processes, represented by squares with different colors. The color scale showed the values (*p*-value).

Kyoto Encyclopedia of Genes and Genomes (KEGG) pathway enrichment was assessed at each time point (12 and 24 hpi), and DEGs were considered significant if they had a corrected *p* value < 0.05. Figure 5 showed an overview of gene regulatory changes in three hosts infected by *P. infestans*. The overview of the comparisons among enriched plant immune-related KEGG pathways showed significant differences among TSHs in responses to infection. The enrichment revealed up-regulated DEGs were significantly correlated with immune activation, while down-regulated DEGs were mainly enriched in carbon metabolism, Biosynthesis of Amino acids and photosynthesis, etc. (*p*-value < 0.05, Figure S4). The TSHs all had activated immune pathways, within which were multiple PTI responses to *P. infestans* infection, such as “MAPK signaling pathway-plant” (sly04016), “plant hormone signal transduction” (sly04075), “phenylalanine metabolism” (sly00360), “phenylpropanoid biosynthesis” (sly00940), and “stilbenoid, diarylheptanoid and gingerol biosynthesis” (sly00945).

In particular, *S. tuberosum* activated many more pathways, including “protein processing in the endoplasmic reticulum” (sly04141), “endocytosis” (sly04144), “autophagy” (sly04136) and “protein export” (sly03060). This result of transcriptional changes in TSHs indicated that *S. tuberosum* had more diversiform response at the early timepoints compared to *N. benthamiana* and *S. lycopersicum* in response to *P. infestans* infection. in the meantime, the speed of infection processing in *S. tuberosum* was faster than that of *N. benthamiana* and *S. lycopersicum*.

### 2.5. Differences in ROS Accumulation of the TSHs

**Figure 6:**
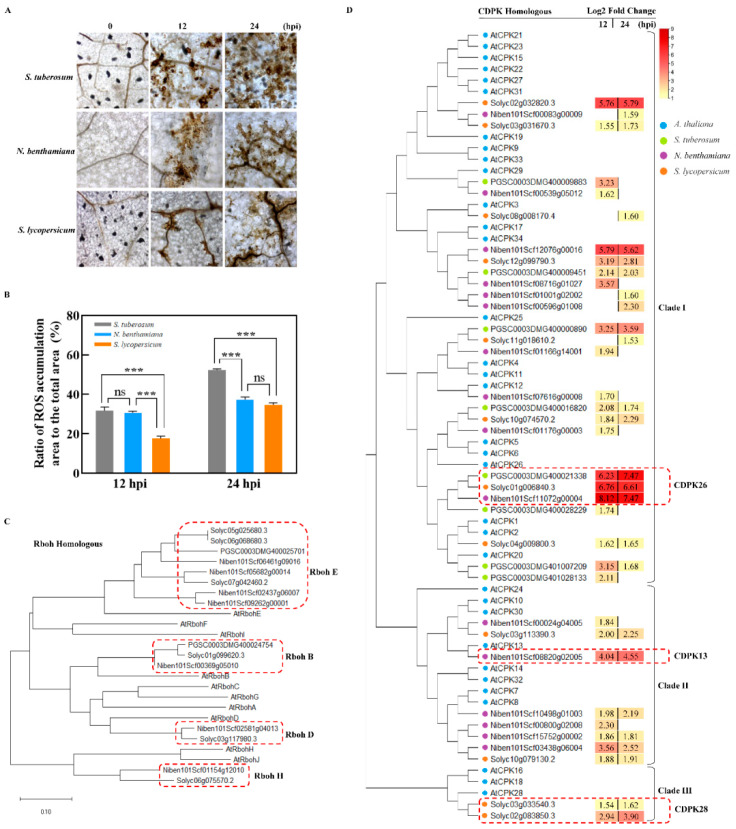
Comprehension analysis of ROS accumulation among the TSHs at 0, 12 and 24 hpi infected by *P. infestans*. (**A**) Phenotype of *P. infestans*-induced ROS accumulation among TSHs. DAB staining for detection ROS burst was performed at 0, 12 and 24 hpi. The dark yellow flecks represented the ROS accumulation sites. Twenty leaves were collected for statistical analysis. (**B**) Quantitative statistics of ROS accumulation ratio to the total area in TSHs at 0, 12 and 24 hpi. Data represent means and standard errors of three biological replicates (ns, *p* > 0.05; ***, *p* < 0.001, one-way ANOVA). (**C**) Phylogenetic tree of respiratory burst oxidase homolog (RBOH) family genes in *A. thaliana* and TSHs. (**D**) Phylogenetic tree of *CDPK* homologous genes in *A. thaliana* and TSHs. Up-regulated genes were selected in transcriptome data used for phylogenetic trees. The rectangle with number represented log2 fold change of *CDPK* genes in TSHs at 12 and 24 hpi. The blue, green, purple and orange dots represent *A. thaliana*, *S. tuberosum*, *N. benthamiana* and *S. lycopersicum,* respectively. The red dotted line boxes indicated to belong the homolog genes, such as *Rboh E*, *Rboh B*, *Rboh D* and *CDPK26*.

Plant recognition of a pathogen activated ROS accumulation that is considered an integral part of the plant defense response and disease resistance induced in plants [24]. To compare the differences in the ROS accumulation level in the different hosts responding to pathogen attack, the infected leaves of TSHs were stained by DAB at 12 and 24 hpi, respectively, and we calculated the ROS accumulation area. Figure 6A showed that TSHs, despite being susceptible, all respond to *P. infestans* infection with a ROS burst. At 12 hpi, focused on the infected spot, the ratio of ROS accumulated area (dark brown) to the total area was 31.68% and 30.67% in *S. tuberosum* and *N. benthamiana,* respectively, which indicated there was no difference in *S. tuberosum* and *N. benthamiana*. That was, however, higher than *S. lycopersicum* with a ratio of 17.65% at 12 hpi. At 24 hpi, that in *N. benthamiana* and *S. lycopersicum* was 37.38% and 34.60% and thus, lower than *S. tuberosum* with 52.35% (Figure 6B).

Based on *A. thaliana* homology, we built a phylogenetic tree of TSH respiratory burst oxidase homolog (RBOH) proteins, which showed that *Rboh* family genes E, B, D and H, each with log2-fold changes ≥1.5 were upregulated in *S. tuberosum*, *N. benthamiana* and *S. lycopersicum*, respectively, at 12 and 24 dpi. Figure 6C showed that the majority of upregulated RBOHs were *RbohE* homologous genes in the TSHs. Indeed, only one *RbohB* gene homolog was specifically induced in each *Solanaceae* species, while no *RbohD* homologues genes was upregulated in *S. tuberosum*.

Furthermore, we constructed a phylogenetic tree of *A. thaliana* homologous TSH CDPK proteins, which showed that those genes were divided into three clades; the majority of the upregulated *CDPK* genes were grouped into clade I in the TSHs (Figure 6D). It is worth noting that those in clades II and III were mainly enriched in *N. benthamiana* and *S. lycopersicum*, with no *CDPK* genes from these clades enriched in *S. tuberosum*. We focused our attention on homologs of *AtCPK26* (PGSC0003DMG400021338; Solyc01g006840.3; Niben101Scf11072g00004) that showed 6- to 8-fold increases in gene expression in all TSHs at both time points, which indicated that those genes could contribute to pathogen stress responses. Furthermore, we focused on *NbCDPK13* (Niben101Scf08820g02005) in *N. benthamiana*; in clade II, it has 4.04- and 4.55- fold up-regulated times at 12 and 24 hpi, respectively. Therefore, we predicted *NbCDPK13* may play an important role in ROS accumulation in *N. benthamiana*. In clade III, only *SlCDPK28* homologous genes in *S. lycopersicum* were up-regulated while there were no up-regulated genes found in *S. tuberosum* and *N. benthamiana.* These uncovered and new candidate genes were possibly involved in the interactions of the *Solanaceae* plants and *P. infestans*.

### 2.6. Differential Gene Expression Related to the Activation of Hormone Signaling Pathways

**Figure 7:**
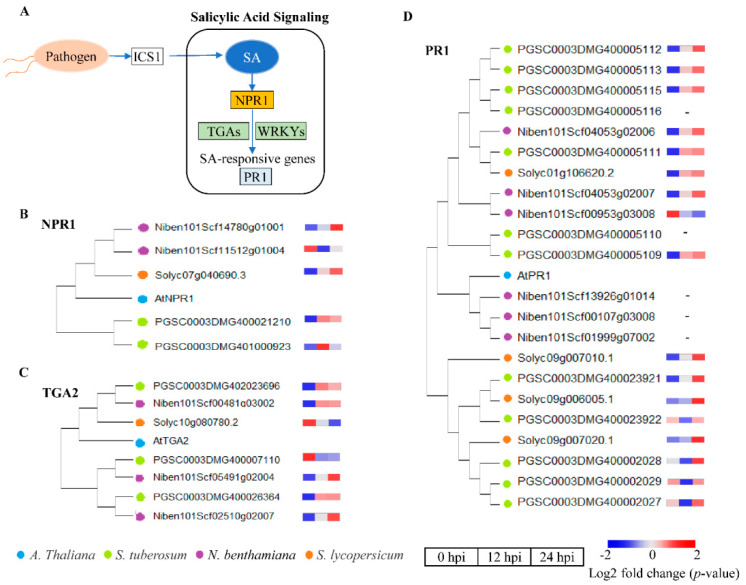
Transcriptional regulation changes of SA signaling pathways related to defense response in TSHs at 0, 12 and 24 hpi infected by *P. infestans*. (**A**) SA signaling pathway was displayed with Key genes in plant. (**B**) The expression of *NPR1* homologous genes in TSHs. (**C**) The expression of *TGA2* homologous genes in TSHs. (**D**) The expression of *PR1* homologous genes in TSHs. The blue, green, purple and orange dots represent *A. thaliana*, *S. tuberosum*, *N. benthamiana* and *S. lycopersicum*, respectively. The rectangles represented the expression levels at 0, 12 and 24 h, respectively. The color scale showed the values of log2 fold change (*p*-value).

Hormone signaling, such as salicylic acid (SA), jasmonic acid (JA) and ethylene (ET), are major plant hormones involved in regulating plant immune responses, plays diverse and critical roles during plant immunity [31,32,33]. In the SA signaling pathway (Figure 7A), the expression of key genes plays an important role in regulation. For example, *NPR1* (DMG401000923), *TGA2* (DMG402023696) and *PR1* (DMG400005112) are marker genes and downstream critical genes of SA-mediated response pathway [33]. Through the construction of phylogenetic tree, the homologous genes of *NPR1* (Figure 7B), *TGA2* (Figure 7C), and *PR1* (Figure 7D) were identified in TSHs and then the expression level of these homologous genes induced by *P. infestans* infection was exhibited. The results revealed that these genes were mostly upregulated at 12 and 24 hpi after *P. infestans* infection among TSHs. This result indicated that *Solanaceae* hosts positively regulated defense functions by activating SA signaling during the process of *P. infestans* infection in the early stage. NPR1 as the direct receptor of SA [39], was induced expressed higher upregulated folds in *S. tuberosum* than *N. benthamiana* and *S. lycopersicum* in 12 h (Figure 7B–D), wherever *TGA* and *PR1* gene of the downstream of *NPR1* showed higher upregulated folds in *S. tuberosum* than *N. benthamiana* and *S. lycopersicum* in 24 h, these results confirmed that the *S. tuberosum* was infected more rapidly than *N. benthamiana* and *S. lycopersicum* by *P. infestans*.

**Figure 8:**
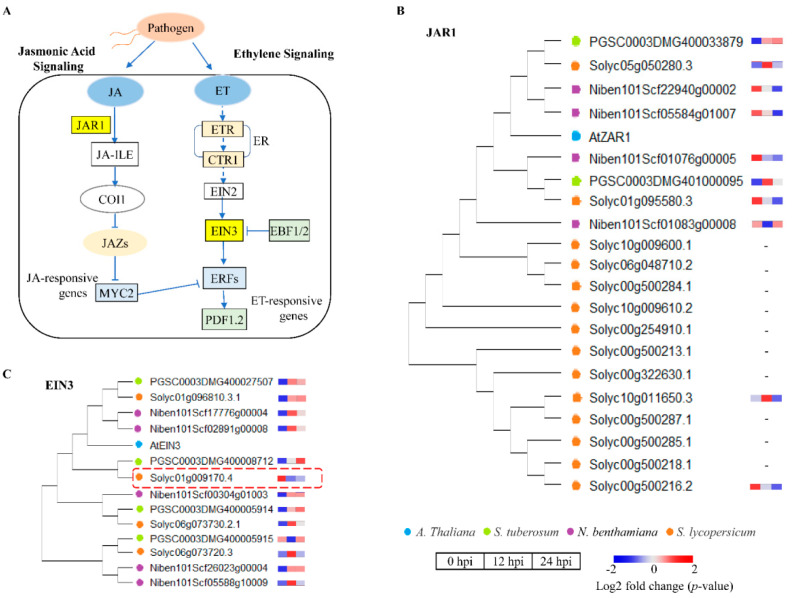
Transcriptional regulation changes of JA and ET signaling pathways related defense response in TSHs at 0, 12 and 24 hpi infected by *P. infestans*. (**A**) JA and ET signaling pathway was displayed with Key genes in plant. (**B**) The expression of *ZAR1* homologous genes in TSHs. (**C**) The expression of *EIN3* homologous genes in TSHs. The blue, green, purple and orange dots represent *A. thaliana*, potato, *N. benthamiana* and *S. lycopersicum*, respectively. The rectangles represented the expression levels at 0, 12 and 24 h, respectively. The color scale showed the values (*p*-value).

Additionally, JA and ET signaling play an important role in plant development and response to a range of biotic or abiotic stress conditions [40,41]. Gene expression analysis of the TSHs showed significant differences in the regulation of the JA and ET signaling pathway. *JAR1* (Jasmonate Resistant 1) is a critical enzyme in the formation of active hormone JA-Ile, which is a key JA signal (Figure 8A). Contrasting expressions of the *JAR1* homologous genes identified in TSHs on phylogenetic tree showed up-regulation in *S. tuberosum*, whereas down-regulation in *N. benthamiana* and *S. lycopersicum* (Figure 8B). As a transcription factors, *EIN3* is the critical components of the ethylene signaling pathway (Figure 8A). Through the construction of phylogenetic tree, 4, 5 and 4 homologous genes of *EIN3* were identified in *S. tuberosum*, *N. benthamiana* and *S. lycopersicum*, respectively (Figure 8C). Based on the expression of these genes, the results indicated that *S. tuberosum* and *N. benthamiana* positively regulated their response functions by activating ET signaling at the early stage of *P. infestans* infection, while complexity in *S. lycopersicum* which displayed both up- and down- regulation. Interestingly, we found *SlEIN3* (Solyc01g009170.4) was down-regulated in *S. lycopersicum*, which may play an important role in regulating its response functions.

### 2.7. Expression of Key Genes and qPCR Verification of the Response of the TSHs to P. infestans Infection

**Figure 9:**
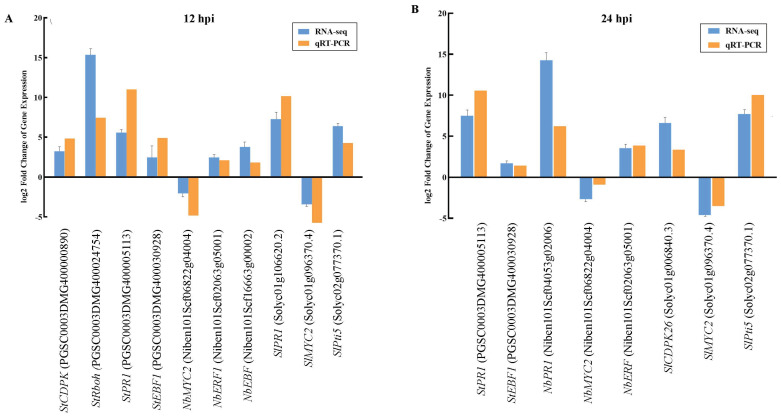
Expression level of marker genes and DEGs obtained by RNA-seq and qRT-PCR validation in potato, *N. benthamiana* and *S. lycopersicum* at 12 (**A**) and 24 (**B**) hpi. Error bars represent standard deviation.

To verify the results of RNA-Seq, the differential expression of key genes involved in ROS accumulation and hormone signaling pathways (SA, JA and ET) in the TSHs that were analyzed based on RNA-Seq, genes were also randomly selected at 12 and 24 hpi and their relative expression level was verified through qRT-PCR. These genes included *CDPK* (PGSC0003DMG400000890), *Rboh* (PGSC0003DMG400024754), *PR1* (PGSC0003DMG 400005113, Solyc01g106620.2), *MYC2* (Niben101Scf06822g04004, Solyc01g096370.4), *ERF1/2* (Niben101Scf02063g05001, Solyc02g077370.1) and *EBF1/2* (PGSC0003DMG 400030928, Niben101Scf16663g00002) at 12 hpi and *CDPK* (Solyc01g006840.3), *MYC2* (Niben101Scf06822g04004, Solyc01g096370.4), *ERF1/2* (Niben101Scf02063g05001, Solyc02g077370.1), *EBF1/2* (PGSC0003DMG400030928) and *PR1* (PGSC0003DMG 400005113) at 24 hpi. The expression levels of all genes were consistent with the results of RNA-Seq at 12 and 24 hpi (Figure 9A,B).

## 3. Discussion

Most plants are resistant to most pathogens, while some are susceptible hosts. The pathogenic lifestyle fundamentally involves feeding from a host [42]. PTI is effective in response to pathogen infection [18,43]. Our DEGs analysis results demonstrated several trends relevant to differences in the timing and level of gene expression in response to *P. infestans* among the TSHs. Previous transcriptome studies have documented foliage defenses against the late blight pathogen are activated in the early stage of TSHs separately [36,44], however, little is known about the differences among the responses of the TSHs to *P. infestans* infection. In this study, we found that the trend is that more genes were expressed in response to pathogen infection in *S. tuberosum* than in *N. benthamiana* and *S. lycopersicum* at 12 dpi. Furthermore, *S. tuberosum* had fewer DEGs and higher gene expression levels than *N. benthamiana* and *S. lycopersicum* at 24 hpi (Figure 3B). Therefore, we found that *S. tuberosum* responding to *P. infestans* was faster and stronger than that of *N. benthamiana* and *S. lycopersicum* in the early infection stage (Figure 1 and Figure 3A). Different inoculation concentrations may influence the timeline of pathogen infection, and thus, the host response [45]. This difference would presumably help with the rapid deployment of response elements derived from immune-induced gene expression. The initiation of infection is a race between the host and pathogen. The pathogen will try to balance the suppression of defense and the spread from the initially infected cell, while the host will try to launch and complete defense actions before the pathogen can do so [46]. We found different gene response models among the TSHs. *S. tuberosum* adopted an “elite troops” strategy (low number of genes, but high intensity of expression), while *N. benthamiana* and *S. lycopersicum* were similar and adopted a “quantity tactics” strategy (high number of genes, but low intensity expression) (Figure 3). Based on the RNA-seq analysis, our research supported the opinion that transcriptional changes in *S. tuberosum* are more pronounced at the early timepoints compared to *N. benthamiana* and *S. lycopersicum* (Figure 4 and Figure 5). Consistent with the gene expression patten, we found similar macroscopic and microscopic evidence of infection difference among the TSHs. Smart et al. had reported that necrosis is seen at the early stages of pathogenesis in *P. infestans*–potato interactions, the interaction between tomato and *P. infestans* can vary considerably, with a more prolonged biotrophic phase, which supported there were different infection time-courses between potato and tomato in response to *P. infestans* [47]. Therefore, those results indicated that different hosts relied on different infection processes under the same time and infection conditions, and the response of *Solanaceous* plants have host specificity while pathogen populations may adapt to host genotypes through increased infection efficiency.

The plant defense system is sophisticated and complex, and it involves many different signaling pathways with extensive cross-talk among them [42]. The oxidative burst, i.e., the generation of reactive oxygen species (ROS) in response to microbial pathogen attack, is a ubiquitous part of the early response mechanisms of plant cells [46]. Plant recognition of a pathogen immediately triggers an oxidative burst, which is considered an integral part of the plant defense response. It has been found that this production of ROS is biphasic: the first phase usually occurs within minutes after pathogen attack but is transient and weak, whereas the second phase is much more intense and sustained, lasting for several hours [42]. In this study, in the DAB staining assay, we found that ROS accumulation in the TSHs could last at least 24 h (Figure 6A,B), which was consistent with previous reports (CUI). Furthermore, we found *S. tuberosum* plants showed the strongest ROS accumulation strength compared with *N. benthamiana* and *S. lycopersicum* plants at 12 and 24 hpi; however, at 12 hpi, ROS accumulation in *S. lycopersicum* plants was slower and weaker compared with that in *S. tuberosum* and *N. benthamiana* plants (Figure 6A,B).

Calcium (Ca^2+^), as important secondary messengers, plays a critical role in signal regulation during plant resistance to pathogen infection through activation and transduction of important downstream immune components (CDPKs, CAM/CML, Rboh, NADPH, etc.) and lead to intracellular biochemical reactions and ROS bursts [28,48]. In this study, we built a phylogenetic tree of *Rboh* and *CDPK* genes from *Arabidopsis thaliana* and the TSHs, of our special interest showed the upregulation of *CDPK* and *Rboh* genes, which are key factors in the activation of ROS [49,50,51,52]. This result provides evidence that the regulation of ROS mediated by key genes in the TSHs in defense reactions against *P. infestans* during plant-pathogen interactions was different (Figure 6C,D). The differences in ROS accumulation among the TSHs indicated that the response speed and process were different as well. Interestingly, we focused our attention on homologs of *AtCPK26* that showed a 6- to 8-fold increase in gene expression in all TSHs at both time points. The results indicated that those genes probably significantly contribute to pathogen stress responses (Figure 6C,D).

Plants have developed a large network of hormonal signaling pathways to cope with pathogen invasion. Among the many plant hormones, SA, JA, and ET are the three major phytohormones released in response to plant pathogens [53,54,55]. Therefore, investigating the signaling and interactions among these three hormones is necessary for understanding the different responses of the three selected *Solanaceous* plants to *P. infestans*. Here, we described the alteration of hormone signaling in the TSHs during *P. infestans* attack. Quantitative analyses suggested that the early activation of some SA-responsive upregulated genes, including *NPR1*, *TGA* and *PR1*, that contribute to an effective defense against *P. infestans* was similar among the TSHs (Figure 7). The reason may be that SA is more effective and sensitive in defending against biotrophic and hemibiotrophic pathogens during the early infection stage. This result indicated that *Solanaceae* hosts positively regulated defense functions by activating SA signaling during the process of *P. infestans* infection. The TSHs could all activate SA signaling, even though JA/ET signaling is more important for resisting necrotrophic pathogens [40,41,54,56]. This result is logical because SA is a quicker and more sensitive signaling pathway during plant-pathogen interactions.

Hormone-regulated factors are able to defend against both biotrophs and necrotrophs. In this study, we found distinct regulation of hormonal signaling among the TSHs. In *N. benthamiana* and *S. lycopersicum*, but not in *S. tuberosum*, we found that JA and ET are significant antagonists of SA through transcriptomic analysis. The reason for this phenomenon may be because of the rapid infection process of *S. tuberosum* (Figure 5). Moreover, incompatible interactions can trigger a series of rapid host defense responses, which includes ROS production, programmed cell death (PCD), etc. [57,58]. EIN3 is the mainstay of ethylene signal transduction, keeping the accurate transmission and execution of instructions, ensuring the orderly transmission of ethylene signals and the healthy growth of the organism. *EIN3* was up-regulated in *S. tuberosum*, probably due to its fast speed of infection process. The difference is that *EIN3* displayed both up- and down- regulation in *S. lycopersicum* exhibiting complexity.

In summary, a fascinating discovery in this study is to provide novel insight into the distinct defense responses of three *Solanaceae* hosts induced by *P. infestans*, and represents a state-of-the-art reference database for the transcriptional regulation of such genes in hosts. We observed the infection progress and ROS burst in this study to demonstrate the timing and level of the pathogen response in the different hosts. These identified DEGs will, in the future, be useful for ascertaining the mode of action of *P. infestans* or elucidating the infection differences among the TSHs. In addition, the data generated in this study have been used to create a gene expression database that is also linked to different Solanum hosts, enabling more accurate decision making in the control of late blight disease in *S. tuberosum* and *S. lycopersicum*. The comparison of the response of these three *Solanaceae* hosts induced by *P. infestans* will allow users to quickly determine expression changes in current *S. tuberosum* and *S. lycopersicum* gene models, resulting in more accurate control of this disease.

## 4. Materials and Methods

### 4.1. Plant Materials

*S. tuberosum* cv. Désirée, *S. lycopersicum* cv. Money Maker and *N. benthamiana* were maintained in the CAU, Peking, China, as the research materials in this study. Potted plants were cultivated in a greenhouse at 24 °C, 60% relative humidity (RH), and a 14 h day/10 h night photoperiod. Vermiculite: peat (1:1) was the substrate in the 8 cm × 8 cm pots used in this study. Leaves from four-week-old potted plants showing good growth and no diseases or pests were used for the inoculation assay and sample preparation for RNA-seq after inoculation with *P. infestans*.

### 4.2. P. infestans Cultivation and Inoculation Assay

The *P. infestans* strain T30-4 maintained in the CAU, Peking, China, was used in this study. The strain was cultured on Rye A agar in petri dishes and cultivated in a constant temperature incubator (PHCbi Co., Ltd., Shanghai, China) at a temperature of 18 °C for 12 days in the dark. Rye A agar medium was made of 1 L distilled water containing 60 g rye extract, 20 g sucrose and 15 g agar. The formation of sporangia was observed under a light microscope, and highly mature sporangia were selected for preparation of sporangia suspension. A moderate amount of pre-cooled (4 °C) ddH_2_O was added to the petri dish, and the surface was scraped with an L-shaped sterile spreader; then, the sporangia suspension was collected in a centrifuge tube. The concentration of sporangia was calculated with a hemocytometer, and ddH_2_O was used to dilute the suspension to 10^5^ sporangia/mL for inoculation of *P. infestans*. A pipettor (Eppendorf China Co., Ltd., Shanghai, China) was used to absorb the sporangia suspension and then dropped onto the plant leaves—10 μL for each inoculation site—and placed them at 18 °C at a moderate temperature ≥80% for infection.

### 4.3. Sample Production and Total RNA Extraction

Leaves of four-week-old potted host plants were inoculated with *P. infestans* at 18 °C for infection and sampled at 0, 12 and 24 hpi; 3 independent replicates of approximately 1 g for each sample were collected. Samples were frozen in liquid nitrogen immediately after collection and transferred to a −80 °C refrigerator for later use. Total RNA was extracted from three biological replicates at each time point using an RNA prep Pure Plant Plus Kit (Zoman Co., Ltd., Beijing, China) according to the instruction manual. A NanoDrop2000 instrument (Thermo Fisher Scientific Inc., Waltham MA. USA) was used to detect the RNA concentration and purity. RNA integrity was detected by 1% agarose gel electrophoresis. RNA integrity was assessed using an RNA Nano 6000 Assay Kit and the Bioanalyzer 2100 system (Agilent Technologies, Santa Clara, CA, USA).

### 4.4. Library Construction and Sequencing

A total of 1 μg of RNA per sample was used for library construction. The initial quantification was performed using a Quit 2.0 Fluorometer. Then, PCR products were purified (AMPure XP system), and library quality was assessed on the Agilent Bioanalyzer 2100 system. After cluster generation, the library preparations were sequenced on an Illumina NovaSeq platform, and 150 bp paired-end reads were generated. Raw data (raw reads) in fastq format were first processed through in-house Perl scripts. Clean data (clean reads) were obtained, and the Q20, Q30 and GC contents of the clean data were calculated. The reference genome published on the *Solanaceae* website was downloaded directly [59]. The index of the reference genome was built, and paired-end clean reads were aligned to the reference genome using Hisat2 v2.0.5. The read numbers mapped to each gene were counted using FeatureCounts v1.5.0-p3, and Fragments Per Kilobase per Million (FPKM) was calculated. Differential expression analysis was performed using the DESeq2 R package (1.20.0) with an adjusted *p* value < 0.05.

### 4.5. GO and KEGG Analysis

The Gene Cluster Profiler R package was used to implement Gene Ontology (GO) enrichment analysis. A *p* value ≤ 0.05 was defined as the threshold for the GO terms, and DEGs that met this condition were defined as significantly enriched. The GO terms in the biological process category were enriched with upregulated genes related to plant immunity. To test the statistical enrichment of KEGG pathways of differentially expressed genes, analysis was performed via alignment to the KEGG database, and the hypergeometric test was applied. The KEGG pathways with *p* value ≤ 0.05 were defined as showing a significant enrichment of DEGs. The KEGG pathways in the TSHs were enriched with pathways related to plant immunity.

### 4.6. Trypan Blue Staining and Microscopic Observation

Observation of the host leaves infected by *P. infestans* was performed using trypan blue staining. Three host leaves inoculated with *P. infestans* for 0, 12, and 24 hpi were immersed in lactophenol trypan blue solution (10 mL phenol, 10 mL glycerol, 10 mL lactic acid, 60 mL absolute ethyl alcohol, 10 mL ddH_2_O, 0.04 g trypan blue (SCRC 710414)). Samples were heated in boiling water for 2 min, and after cooling, they were heated for another 2 min. The samples were soaked in the staining solution overnight. The samples were transferred to chloral hydrate solution (250 g chloral hydrate (SCRC 300375), 100–150 mL ddH_2_O), incubated at room temperature and destained overnight. Then, the samples were observed under a microscope and imaged.

### 4.7. Quantification of Cell Death Intensity

Firstly, the color mode of the image was changed to 8-bit grayscale image by ImageJ software, and then the pixel size of the image was converted to the actual physical size (mm). The automated threshold was used to invert the whole grayscale image into a black and white image. The black area was determined. Each host 30 infected spots collected that were counted for statistics using GraphPad Prism 8 software.

### 4.8. Relative Biomass of P. infestans Determination Assay

Total DNA was extracted from diseased leaves. Each sample was mixed with 20 inoculation leaf discs and repeated three times. *P. infestans Actin* gene was used as a detection gene and plant *Actin* genes were used as reference genes. PCR was performed in 50 μL reactions with *PiActin* primer. Each PCR cycle included denaturation at 94 °C for 30 s, annealing at 56 °C for 30 s, and elongation at 72 °C for 30 s. The PCR products were analyzed on a 1.5% agarose gel and stained with ethidium bromide. The grayscale of Actin DNA band was measured from agarose gel bands after transformation quantified by ImageJ software. Relative biomass was calculated by the ratio of Actin DNA of *P. infestans*/plant tissue.

### 4.9. Reactive Oxygen Species Accumulation Assay

ROS accumulation in the infected leaves of the TSHs was detected by diaminobenzidine (DAB) staining assay [60]. Leaves of the TSHs inoculated with *P. infestans* at 10^5^ sporangia/mL were immersed in 1 mg/mL (*w*/*v*) DAB solution (0.1 g DAB, 100 mL ddH_2_O, 0.1% Tween 20, pH = 3.8), stained at 25 °C for 8 h in the dark, collected at 12 h and 24 h after inoculation, and destained with 95% ethanol for at least 10 h before imaging. The area of ROS accumulation was calculated by ImageJ software with reference to 4.7. Statistical analysis was conducted using SPSS 22.0 software (SPSS Inc., Chicago, IL, USA). The ratio of the accumulated area to the total area was analyzed using Tukey’s test at the 0.05 probability level to assess the significance. The descriptive statistics are shown as the mean values and standard errors of the mean.

### 4.10. Real-Time qRT–PCR Assay

The infected leaves were collected according to the previously described method for RNA-seq sample preparation, and RNA was extracted. A total of 1 μg of RNA per sample was used for reverse transcription using the PrimeScript RT Kit (Takara Biotechnology (Dalian) Co. Ltd., Dalian, China). Then, AceQ^®^ qPCR SYBR Green Master Mix (TransGen Biotech Co., Ltd., Beijing, China) was used for qRT–PCR assays with a Bio–Rad CFX96 System. The primers employed for qPCR were designed with the NCBI Primer design tool [61]. Primer sequences for qRT–PCR are listed in Table S3. Relative expression was calculated using the 2^−ΔΔCt^ method. Three independent biological replicates were used for each sample.

## Data Availability

The raw sequencing data in this article are stored in the CNGB Sequence Archive (Accession number CNP0002222).

## Supplementary Materials

The following are available online at https://www.mdpi.com/article/10.3390/ijms222011000/s1.
