# Supplementary material for: Comparison of the Distinct, Host-Specific Response of Three Solanaceae Hosts Induced by Phytophthora infestans
Source: Int J Mol Sci. 2021 Oct 12;22(20):11000. doi: 10.3390/ijms222011000 (PMC8537708; doi:10.3390/ijms222011000)
Supplement: Supplementary file 1 [file ijms-22-11000-s001.zip › Fig S1Phenotypes of infection on TSHs .pdf]

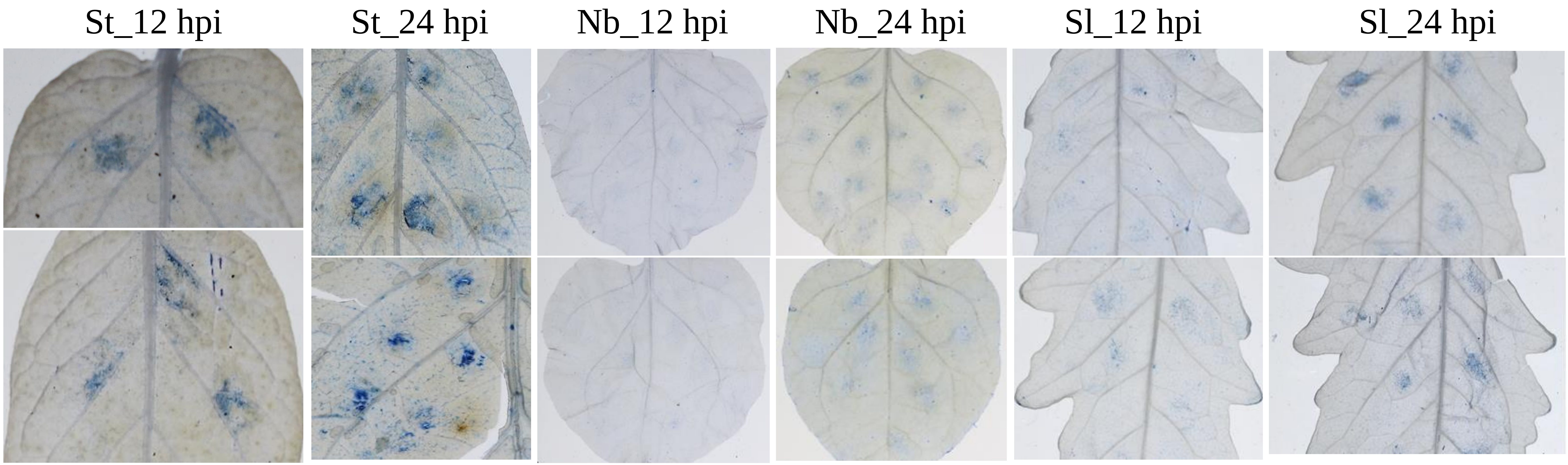

Fig S1: Phenotypes of infected leaves on TSHs at 12 and 24 hpi

Comparisons of *P. infestans* infection on the leaves of TSHs were confirmed by macroscopic observation at 12, 24 hour post inoculation (hpi). At 12 dpi, small light blue spots stained by typan blue were observed on *S. tuberosum* leaf, but only very small and few black spots could be macroscopically observed on the leaf of *N. benthamiana* and *S. lycopersicum* compared to *S. tuberosum*. The infection site turned to dark blue stained by typan blue, obvious damage could be observed on *S. tuberosum* leaf at 24 dpi. At 12 hpi, *P. infestans* infection was observed in cells of the TSH.
