# Supplementary material for: Comparison of the Distinct, Host-Specific Response of Three Solanaceae Hosts Induced by Phytophthora infestans
Source: Int J Mol Sci. 2021 Oct 12;22(20):11000. doi: 10.3390/ijms222011000 (PMC8537708; doi:10.3390/ijms222011000)
Supplement: Supplementary file 1 [file ijms-22-11000-s001.zip › Fig S2 observation.pdf]

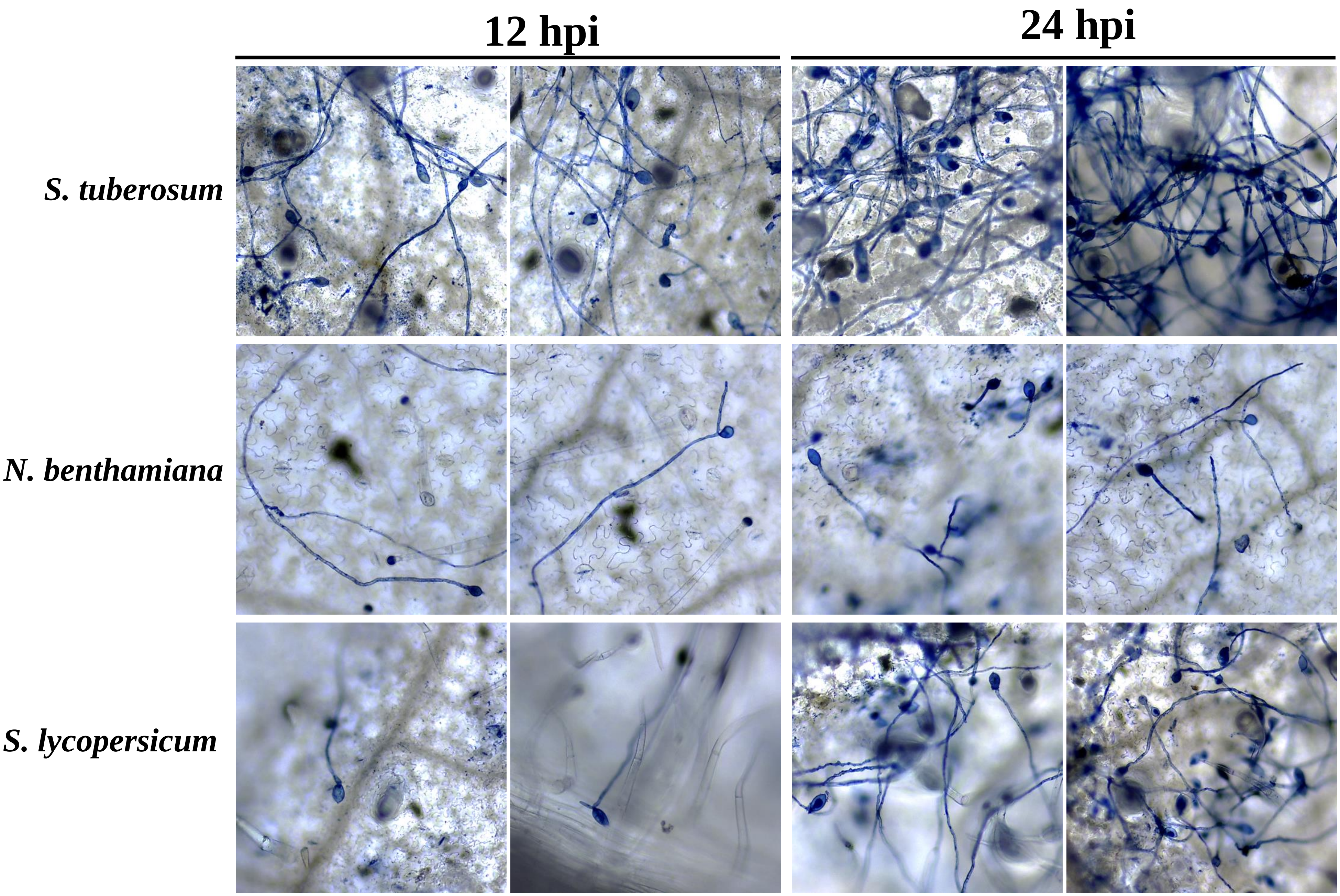

Fig S2. Phenotypes of P. infestans on TSHs at 12 and 24 hpi

Comparisons of *P. infestans* infection on the leaves of TSHs were confirmed by microscopic observation at 12 and 24 hour post inoculation (hpi). At 12 hpi, *P. infestans* infection was observed in cells of the TSH. Young hyphae broadly spread on the leaves of TSHs, *N. benthamiana* showed the fewest hypha compared to *S. tuberosum* and *S. lycopersicum* at 24 hpi. More numbers of hyphae were microscopically observed on TSHs' leaves by microscopic observation compare to 12 hpi.
