# Supplementary material for: Comparison of the Distinct, Host-Specific Response of Three Solanaceae Hosts Induced by Phytophthora infestans
Source: Int J Mol Sci. 2021 Oct 12;22(20):11000. doi: 10.3390/ijms222011000 (PMC8537708; doi:10.3390/ijms222011000)

Figure S4. Top ten KEGG pathways enrichment with down-regulated genes of *S. tuberosum*, *N. benthamiana* and *S. lycopersicum* at 12 and 24 hpi

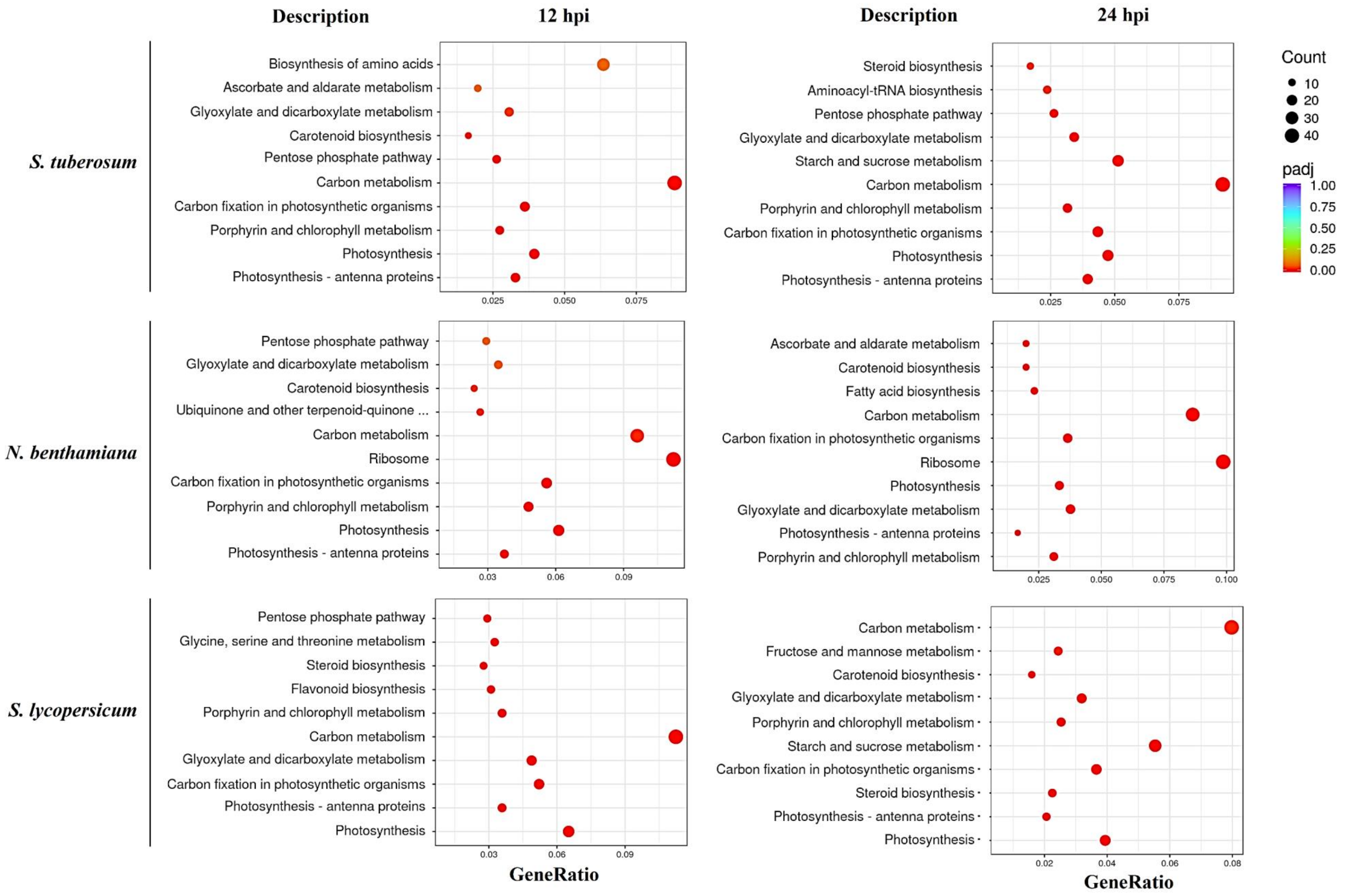

Supplement: Supplementary file 1 [file ijms-22-11000-s001.zip › Fig S4.pdf]
