# Supplementary material for: Comparison of the Distinct, Host-Specific Response of Three Solanaceae Hosts Induced by Phytophthora infestans
Source: Int J Mol Sci. 2021 Oct 12;22(20):11000. doi: 10.3390/ijms222011000 (PMC8537708; doi:10.3390/ijms222011000)
Supplement: Supplementary file 1 [file ijms-22-11000-s001.zip › FigS3 biomass.pdf]

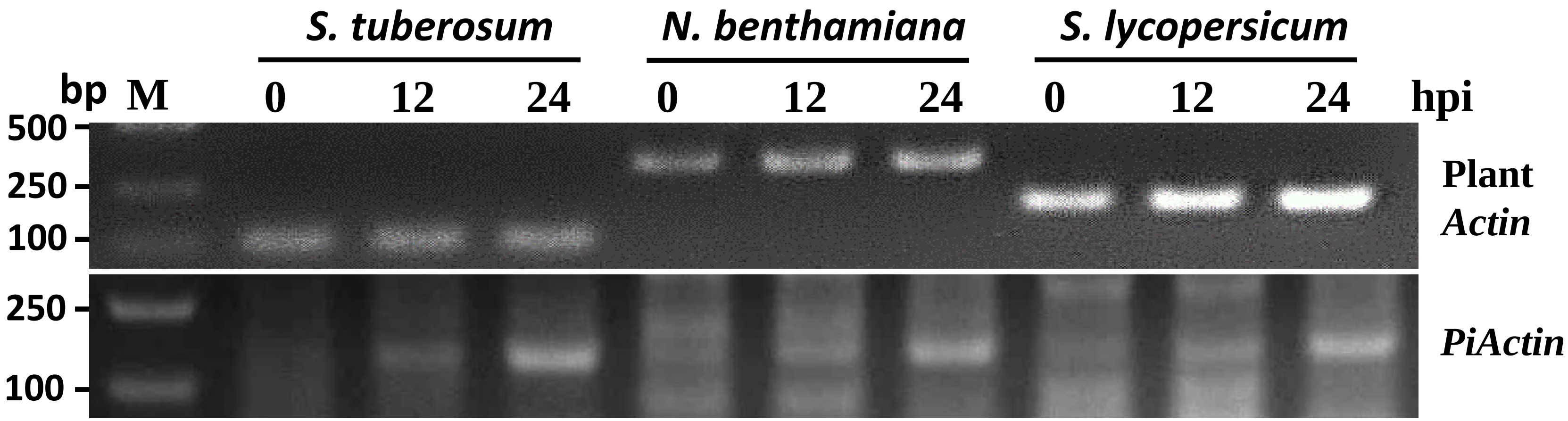

Fig S3 Evaluation Actin genes of plant and *P. infestans* in plants infected with *P. infestans*。

Relative biomass of *P. infestans* from diseased leaves in TSHs at 12 and 24 hpi via semi-quantitative amplification. Sample was mixed with 20 inoculation spots. M, Takara DL marker 2000.
