# Supplementary material for: Comparison of the Distinct, Host-Specific Response of Three Solanaceae Hosts Induced by Phytophthora infestans
Source: Int J Mol Sci. 2021 Oct 12;22(20):11000. doi: 10.3390/ijms222011000 (PMC8537708; doi:10.3390/ijms222011000)
Supplement: Supplementary file 1 [file ijms-22-11000-s001.zip › Table S1 basic information.pdf]

Table S1. Basic Information on Transcriptome Data

Samples	Time Points	Total Reads	Q20 Percentage (%)	Q30 Percentage (%)	Mapped Reads	Mapping Rate (%)
St_0_1	0 h	78416786	97.26	92.43	69526678	88.66%
St_0_2	0 h	70426770	97.42	92.74	62607068	88.9%
St_12_1	12 h	73246444	97.71	93.37	62316420	85.08%
St_12_2	12 h	73955992	97.59	93.13	62045128	83.89%
St_24_1	24 h	76644112	97.3	92.54	61450231	80.18%
St_24_2	24 h	87852282	97.06	92.01	62075417	70.66%
St_24_3	24 h	81779604	97.21	92.37	64367473	78.71%
Nb_0_1	0 h	84504674	97.38	92.71	81996501	97.03%
Nb_0_2	0 h	77896360	98.31	94.82	75256738	96.61%
Nb_12_1	12 h	80982710	97.29	92.52	75336816	93.03%
Nb_12_2	12 h	75132718	97.33	92.53	72369100	96.32%
Nb_12_3	12 h	82114838	97.19	92.26	77337181	94.18%
Nb_24_1	24 h	72701090	97.28	92.44	65459677	90.04%
Nb_24_2	24 h	80920586	97.36	92.66	71022033	87.77%
Nb_24_3	24 h	73505016	97.4	92.79	57223615	77.85%
Sl_0_1	0 h	73411528	97.26	92.44	70733424	96.35%
Sl_0_2	0 h	68786988	98.27	94.61	66620574	96.85%
Sl_12_1	12 h	76611550	97.43	92.8	73512802	95.96%
Sl_12_2	12 h	1.01E+08	97.58	93.1	97743010	96.43%
Sl_24_1	24 h	81660754	97.4	92.74	78953596	96.68%
Sl_24_2	24 h	70491498	97.54	92.99	67228769	95.37%
Sl_24_3	24 h	78998802	97.36	92.63	72451927	91.71%

Q20 percentage: The ratio of nucleotides with quality value ≥ 20; Q30 percentage: The ratio of nucleotides with quality value ≥ 30; Mapping rate: The percentage of mapped reads of the total high quality, filtered reads. St: *S. tuberosum*; Nb: *Nicotiana benthamiana*; Sl: *S. lycopersicum*.
